# Practical applications of gamification in patient-centered outcomes research and digital health, and its acceptance in clinical trials

**DOI:** 10.3389/fdgth.2026.1652217

**Published:** 2026-05-29

**Authors:** Christopher Hartford, Roland Barge, Tenage McDowell, Beth Gentsch, Tara Symonds, Diana Rofail

**Affiliations:** 1 Regeneron Pharmaceuticals, Inc., Sleepy Hollow, NY, United States; 2 Clinical Outcomes Solutions, Folkestone, Kent, United Kingdom; 3 Washington University in St. Louis, St. Louis, MO, United States

**Keywords:** clinical trials, education, e-learning, game-based learning, gamification, patient experience, site experience

## Abstract

**Background:**

The application of game elements to engage participants and improve data collection for clinical trials is relatively novel, with limited research around the impact of gamification in clinical research. This article explores published literature and surveys from patients and clinical sites.

**Methods:**

A targeted literature review was completed in November 2025 to identify published articles (≤10 years) on the application of gamification in clinical trials. Synthesized findings informed the design of two surveys of US adults (*n* = 1,044 from UserTesting.com) and clinical trial sites (*n* = 311) on their perceptions of gamification and acceptance in clinical trials. Both were ∼5-minute online surveys utilizing five open- and closed-ended questions.

**Results:**

Twenty-four articles were focused on the application of gaming design and mechanics to non-gaming activities. Three primary areas identified were education (*n* = 3), health outcomes measures (*n* = 7), and patient engagement (*n* = 14). Eighteen studies reported an advantage of gamification, including positive impacts on health outcomes measures (*n* = 5) and patient engagement (*n* = 11). Survey respondents (adults) were most familiar with computer games (62%), stating a preference for participating in trials that included gamified cell phone applications, with the ability to customize application elements as the most important. From a thematic analysis of respondents’ comments, potential impacts on human behavior and performance (33%) were the most prevalent concerns. Data (26%; including concerns about privacy, integrity, and security), and software (22%; including adaptability to account for ability and skill variation, satisfaction, user experience, controls, customization, and personalization) were also key areas of concern for patients. Key perceived benefits included improvements in experience (31%) and engagement (24%). Site respondents were most familiar with managing gamified clinical trials with gamified cell phone applications (30%) and would prefer to manage trials that included these elements vs. traditional trials. Notifications, education, and training were the most important gamification elements for site respondents.

**Conclusions:**

Potential advantages of gamification include increased engagement, trial education, adherence to protocols, and enjoyment of the clinical trial experience, which may increase retention and data completeness. Further research is required to better understand the potential impact of gamification on scores of how patients feel or function.

## 1 Introduction

Gamification, the application of gaming design and mechanics to non-game environments or activities, has increasingly permeated various fields including education, marketing, and healthcare. Its primary aim is increasing user engagement or motivation towards a certain behavior, as well as aiding the acquisition of new knowledge, attitudes, or skills, via rewarding, competition, or personalization (1). The proliferation of new technologies, widespread internet access, social media, and surge in smartphone ownership (2) have opened innovative avenues for health agencies and researchers to connect with users (3). In the context of clinical trials, researchers continue to investigate how the elements of gamification, such as rewards, competition, and personalization, can be leveraged to boost user engagement and motivate behaviors like increased activity or medication adherence (3–6).

The potential of gamification to enhance user engagement and motivation is well-documented. For example, a review of empirical literature conducted by Hamari et al. in 2014 found that gamification, when applied in various contexts including commerce, education, health, work, and data-gathering, can increase user activity and provide motivational affordances, encouraging desired behaviors (7). However, the effectiveness of gamification is highly context-dependent, which is particularly relevant in clinical trials where participant engagement is crucial for data integrity and study success. Previous research has unearthed various motivational affordances offered by gamification, such as points, leaderboards, achievements/badges, levels, stories/themes, clear goals, feedback, rewards, progress, and challenges, hereafter referred to as “gamification elements.”

Games are motivating due to their cognitive, emotional, and/or social appeal (8). Cognitive processes are particularly critical to the effectiveness of gamification for enhancing user engagement and learning, where, for example, gamification elements such as points, feedback, and rewards have been evidenced to enhance core cognitive functions such as attention and memory (9). By leveraging its influence on cognitive processes and its motivational affordances, gamification has the potential to enhance user participation.

When translated to the healthcare setting, gamified electronic health interventions have been associated with improved adherence to medication regimens (10), whereas leaderboards, badges, and progress tracking have been associated with enhanced self-management and improved health outcomes (11). In both instances, however, positive effects of gamification were short-term and warranted further study to better understand the emerging use of gamification. Leveraging game-design elements may offer opportunities for enhanced participant engagement, adherence, or data quality.

Carefully implemented gamification may hold an innovative role in increasing engagement in large-scale clinical trials (12). Recent advances in technology, including its availability, its relevance to decentralized trials, and its potential for electronically implemented methods of data collection, interaction with staff (e.g., remote visits, or remote data entry and monitoring), and procedures for clinical trials, offer great opportunities to promote more enjoyable electronic interfaces, which may thus increase engagement with clinical trials.

Despite emerging gamification data, and the increase in the availability of technology, the application of gamification in clinical trials remains limited. This study therefore aims to build on previous findings by exploring the perspectives of participants and clinical trial sites to inform strategies for effectively implementing gamification in future clinical trials.

## 2 Methods

This research was conducted in two parts: (1) a review of examples of gamification in existing literature; and (2) two surveys designed to understand the current level of acceptance of gamification within a clinical trial context from the perspectives of both participants and clinical trial sites, and to expand upon literature review findings.

### 2.1 Targeted literature review

#### 2.1.1 Objective

This review was descriptive rather than systematic, given the research question and diversity in study designs. Our aim was to provide practical insights for stakeholders primarily involved in clinical trials, including trialists and site personnel. The literature review served as a foundation for evidence consolidation which was complemented by surveys capturing stakeholder perspectives on gamification benefits in clinical trials.

The primary objective of the targeted literature review was to explore the role of gamification in clinical trials, with the specific aim of addressing the following questions:

1. What is the definition of gamification in the context of clinical trials?
2. How have gamification elements been applied in the context of clinical trials (e.g., training, patient engagement/retention)?
3. What is the impact of gamification on how patients feel, function, and survive as measured by different types of clinical outcomes assessments (COAs) within clinical trials?

Evidence from the literature review was summarized descriptively using a data-extraction table. Findings from the review allowed a more granular understanding of the advantages, challenges, and/or gaps within the use of elements of gamification in clinical trials, with the intent of exploring identified themes further in subsequent stages of the research.

#### 2.1.2 Search strategy

The literature review was completed in November 2025, using OVID to search the Medline, EMBASE, and PsycINFO biomedical research databases to identify articles involving aspects of gamification elements. The following search terms were used: “(patient reported outcome* or patient engagement or clinical outcome* assessment* or COA* or caregiver report* or observer report* or clinical trial*) AND (*gamification*).” To identify the most relevant articles, search terms were limited to abstracts and titles of publications, research involving humans, published in English, and published within the last 10 years (Table 1).

**Table 1:** Targeted literature search strategy.

OVID (EMBASE, Medline, PsycINFO)		
Search #	Search terms	Returns
1	(patient reported outcome* or patient engagement or clinical outcome* assessment* or COA* or caregiver report* or observer report* or clinical trial*).ab,ti	3,961,889
2	(gamification).ab,ti	5,043
3	1 AND 2	440
4	Limits: Humans AND English AND published within last 10 years	231
5	Deduplicate	177
Other sources		
Google Scholar	Gamification and clinical trials	16,800
Clinicaltrials.gov	Gamification [Phase 2, 3, 4]	2

Literature search 1 was conducted on August 12, 2022. Literature search 2 was conducted on November 06, 2025.

COA, clinical outcomes assessment.

A review of Google Scholar and ClinicalTrials.gov supplemented the findings from the OVID review. Articles identified within the first 10 pages of results from the Google Scholar search engine were reviewed by title, abstract, and full text before proceeding to data extraction (Table 1).

All peer-reviewed articles, websites, or clinical trial records that reported the use of gamification in clinical trials within the past 10 years were included in the data extraction. Articles were excluded if they did not discuss gamification or its potential impact on clinical trial design, the patient experience, patient recruitment/retention, or data capture using COAs. Articles were also excluded if they were duplicates or if they were not published in English.

#### 2.1.3 Data extraction

The literature review results were screened using the PRISMA method (13). The research team agreed on *a priori* rules to extract the following information into a descriptive summary table: article information (author, objectives, design, and results), gamification definitions, motivational affordances (methods/elements of gamification utilization) (7), and results (e.g., impact on trial engagement/performance or on how patients feel and function). Following review of the data and/or commentary from each article, the research team assigned a label to each study to indicate whether the gamification element was associated with a positive (e.g., an observed increase in engagement) or negative impact. Based on data extraction findings, each paper was organized into higher-order areas of application. Findings from the literature review were descriptively summarized.

### 2.2 Surveys

#### 2.2.1 Objective

Results from the literature review gathered evidence expanding the current understanding of the use of gamification in the healthcare setting. The review results formed themes explored in two surveys: one of US adults and one of representatives worldwide from clinical trial sites. The surveys investigated the current familiarity of participants and clinical trial sites with elements that were reported within the literature review. The surveys also identified perceptions of, acceptance of, and opportunities for, the use of gamification. The topics surveyed were gamified mobile applications, computer games, augmented reality, virtual reality (VR), and other gamified elements (notifications, points, badges, challenges, collaboration capabilities, avatars, storylines, videos, audio, haptic experiences, and training/education). Survey data were analyzed to understand both the potential benefits and challenges of gamification from each perspective.

#### 2.2.2 Survey development and administration

Both surveys were developed by the Regeneron User Experience expert in accordance with a study protocol. Both surveys underwent two rounds of review, including one round of review by cross-functional teams (digital biomarkers, development innovation, and digital health technology), followed by one round of review by subject matter experts (patient centered outcomes research and digital health) prior to finalization to ensure survey understandability and appropriateness for the research objectives.

Both surveys were administered online and utilized a combination of response options, including free-text responses for open-ended questions as well as single/multiple select, ranking, and Likert-type response scales. Each survey was completed once and took around 5 min to complete. No respondents’ identities were disclosed, and data for each participant was imported into Microsoft Excel by participant ID number to maintain participants’ anonymity.

Due to the nature of the survey respondents, i.e., US adults and clinical site staff who had previously participated in Regeneron Pharmaceuticals, Inc. clinical trials, the surveys were classified as market research. As such, institutional review board approval was not sought.

#### 2.2.3 Participants

Participants between 18 and 90 years of age were included if they had previously provided informed consent, joined the UserTesting.com (formerly UserZoom) respondent panels, and were able to understand and complete study-related questionnaires. UserTesting.com respondent panels consist of a global network of participants who have opted-in to provide feedback on experiences, products, and more.

#### 2.2.4 US adults survey

UserTesting.com was used for the US adult survey. This platform is a type II certified and General Data Protection Regulation–and Health Insurance Portability and Accountability Act–compliant platform, allowing surveys to be rapidly distributed to US adults who have pre-consented to provide survey responses on any topic (14). The gamification survey was targeted at 1,000 US adults as a primary key market area in August 2023.

Respondents were provided with the following information and then asked to respond to a short series of questions: “Imagine that you have been asked to take part in a clinical trial that is studying a potential medicine that may improve a serious health condition or disease. As part of this clinical trial there is a selection of new wearable devices and software applications that will allow researchers to continuously monitor your health and ask you questions using a survey application on your cell phone or tablet.”

Adults were advised before the start of the survey that no payment would be provided by the survey requestor, and were asked to confirm they understood that their anonymized responses would be collected, analyzed, shared, and published.

The survey included an initial question asking whether participants were healthy or lived with an illness, while the remaining five questions topics included:

- Familiarity with popular types of gamification (closed response).
- Preferences for participating in trials containing a gamified element (closed response).
- Importance of listed gamification elements as part of a gamified clinical trial experience (closed response).
- Suggestions for gamification approaches (open-text response).
- Main benefits and concerns of using gamification in clinical research (open-text response).

#### 2.2.5 Clinical trial sites survey

For the clinical trial site survey, 1,000 sites from Regeneron’s existing database of worldwide clinical trial sites were contacted. Prior permissions allowed for re-contact via email and data collection for future market research. The contacts were imported into an online survey platform (SurveyMonkey), and a survey similar to that sent to the US adults was sent to explore sites’ perceptions of gamification and their acceptance of its use in clinical trials. Sites were provided with contextual information and asked to respond to a short series of questions; they were invited to participate regardless of their specificity to any therapeutic area or disease specialty, with the intent to capture data broadly and agnostically.

Sites were asked to imagine that they had been asked to manage a clinical trial that included a selection of new devices and software applications that would allow them to continuously monitor patients’ health in various ways as well as ask questions using an application on a cell phone, smartphone, or tablet.

Full details of the questions and response options for both US adults and trial site respondents are provided in the Supplementary Table S1.

#### 2.2.6 Analysis of survey responses

Responses to each question within both surveys were quantified in terms of rank and frequency. Where relevant, qualitative feedback from respondents was also provided to aid interpretation of the rankings and frequencies. Each rank value (*V_i_*) was assigned a number from 1 (not at all important) to 5 (extremely important), with respondents choosing one rank per element of gamification. The frequency of each interval was calculated by multiplying the number of respondents (*N_i_*) selecting a rank by the rank value (e.g., 1 × 16 for 16 participants endorsing a rank of “not at all important” for a single gamification element). Scores for the different rank values were then summed (*R*) for each gamification element to generate the overall rank score. This approach allowed for an understanding of the overall importance assigned to each of the elements by respondents.

The rank score calculation was:

$$R=\overset{5}{\underset{i=1}{\sum }}(V_{i}*N_{i})$$

Open-text responses were analyzed thematically. The first step involved familiarization with the data, which entailed reading the responses to understand the feedback obtained, and participant feedback was then codified thematically. Feedback provided by different participants on the same topic was thematically combined under a single label within Excel (e.g., “data privacy” was coded to sections of text where participants described concerns about the security and privacy of the data collected via gamification elements). The codes were then reviewed, and similar codes were analyzed to identify broader patterns, which informed the development of higher-order themes. For example, the code “data privacy” was grouped into the broader theme of “data”. Themes were reviewed and modified as needed to ensure they reflected the nature of the participant responses and related back to the research objectives. The frequency of responses was also quantified and categorized into low, medium, and high frequency per code. Additionally, sentiment analysis was conducted whereby the emotional tone of open-text responses was evaluated. Each response was labeled as positive, neutral, or negative. Findings were investigated both individually by survey type and compared side by side.

Subgroup analyses were conducted to investigate differences among demographics and site characteristics. US adult survey responses were divided into age categories (18–24, 25–34, 35–44, 45–54, and ≥55 years), and trial site survey responses were divided into type of site (private practice, private/community hospital, dedicated research clinic, and academic medical center). To enable comparability, rank scores (*R*) were transformed into an average rank score (*A*) by dividing the rank score for each subgroup by the size of the subgroup (*n*):

$$A=\frac{\sum_{i=1}^{5}(V_{i}*N_{i})}{\sum_{i=1}^{5}N_{i}}$$

## 3 Results

### 3.1 Targeted literature review

**Figure 1:**
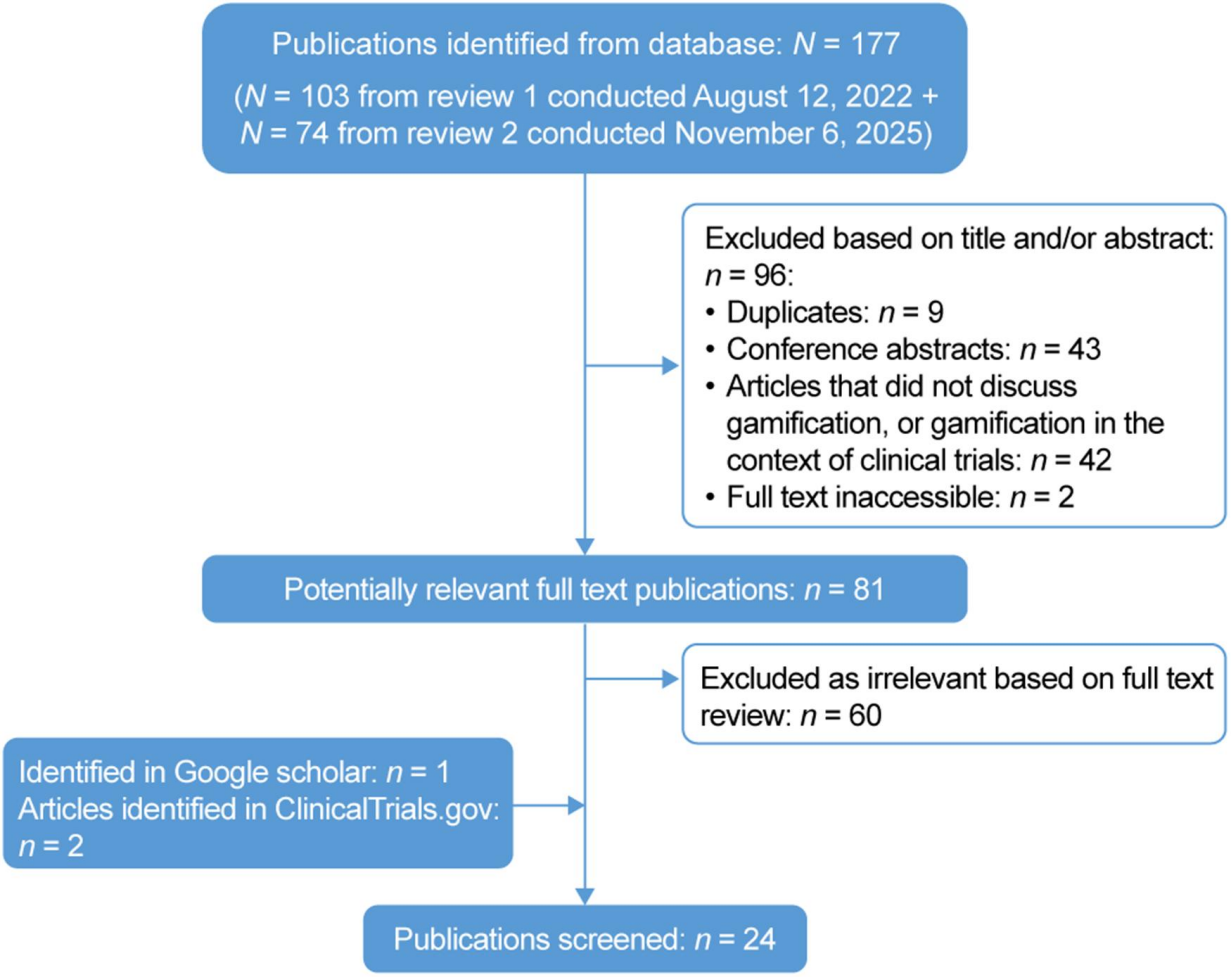
PRISMA diagram for OVID literature review. OVID includes EMBASE, Medline, and PsycINFO.

A total of 177 publications were identified from the OVID literature search. Of these, 172 were screened (five were duplicates) first by title and abstract (*n* = 96 excluded at this stage) and then by full text (*n* = 60 excluded at this stage). A final 22 articles met the prespecified eligibility criteria and were reviewed and summarized (Figure 1). An additional article was identified from Google Scholar, along with two publications identified from ClinicalTrials.gov; a total of 24 full-text articles were therefore included in the data extraction.

The gamified approaches identified in the literature ranged from small gamification elements such as leaderboards to full immersion in VR. Three primary categories of gamification were identified from the literature: education (*n* = 3); measurement of health outcomes (*n* = 7); and patient engagement (*n* = 14) (Supplementary Table S2) (1, 3, 6, 15–34). Eighteen of the 24 studies suggested that gamification had a positive impact on either patient education (*n* = 2), the measurement of health outcomes (*n* = 5), or increasing patient engagement (*n* = 11).

The articles identified in the review reported on different aspects of gamification utilization within studies of various designs, including:

- Randomized controlled trials (RCTs; *n* = 16/24)
- Non-RCTs (*n* = 1/24)
- Non-controlled prospective studies (*n* = 3/24)
- Systematic review (*n* = 4/24)

The studies which found a positive effect on health outcomes and/or patient engagement were predominantly RCTs utilizing digital intervention (gamification) groups vs. control (traditional approach) groups aimed at enhancing physical activity, engagement, knowledge, or adherence. Of the five studies that did not posit findings in favor of gamification, most were RCTs (similar to the positive studies), but did not report notable improvements in relevant patient outcomes [knowledge gained, post-operative outcomes, physical activity, weight loss, anxiety, panic, stress, and quality of life (QoL)]. Though there were no clear trends, longer follow-up periods, more complex/engaging elements of gamification (such as customization/personalization and missions/challenges), and incorporation of social interactions (such as team-based challenges or competition) were apparent in studies reporting positive gamification evidence. Information on the articles identified from the literature review is summarized in Supplementary Table S2 and the sections that follow.

#### 3.1.1 Definition of gamification in the context of clinical trials

The definition most often employed for gamification was the application of gaming design and mechanics to non-gaming environments or activities (identified in 9 of 24 articles) (1). Additionally, gamification was described as taking gaming elements of rewards, competition, and personalization to engage participants in clinical trials and motivate them toward preferred behaviors (3). Game based interventions, such as game-based exercise interventions, were sometimes referred to as “serious games”. These interventions often had the goal of behavioural change i.e., the use or gain of new knowledge, attitudes, or skills (29).

#### 3.1.2 Elements of gamification identified

Several elements of gamification were identified from the literature, and most studies used multiple gamification elements. The most frequently employed elements were points, levels, leaderboards, badges, rewards, challenges, feedback, narrative structures, personalization, and social connectivity, which were divided into four main categories: notifications (e.g., to complete an activity and track progress), rewards and incentives (e.g., to encourage completion of activities and goals), multi-modal interactions (e.g., apps, sensor-based technologies with movement, and voice recognition), and education.

Notably, points, rewards, and progress tracking were consistently associated with increased short-term engagement, while social features such as leaderboards and team-based competition were intended to enhance participation. Feedback and personalization contributed to user satisfaction. Details on gamification elements identified are described in the following sections and are provided in Supplementary Table S2.

#### 3.1.3 Education about clinical research participation

The review identified that games are used to provide information about clinical trials, in order to encourage participation. For example, the Paper Kingdom Game is a video game designed to teach children aged 8–14 years about confidentiality, withdrawal rights, and the importance of clinical research, through a simulated rescue mission where players defeat dragons representing research fears (12). One study compared knowledge of clinical trials between children who played the Paper Kingdom Game and those who were supplied with a paper information sheet, revealing that although all users of the game increased their knowledge, the group supplied with the paper information sheet showed greater knowledge about clinical trials (12).

#### 3.1.4 Measurement of health outcomes and potential for impact on how patients feel and function

Two articles utilized exergames (e.g., Microsoft Kinect) to measure physical and/or cognitive status while encouraging participant exercise (28, 29). One study was conducted in patients with shoulder impingement syndrome receiving arthroscopic subacromial decompression, where one group received Kinect-aided physiotherapy and another followed a standard physiotherapy protocol. In this study, Oxford Shoulder Score and Disability of the Arm, Shoulder, and Hand ratings showed a significant improvement in the standard protocol group but not in the gamified group (28).

Another study investigated gamification in three groups of hospitalized older adults: one received a gamified activity diary to document daily completion of physical and cognitive activities via a walking trail and culture quiz; the second received supervised physical and cognitive training using the Health Arcade prototype (VR); and the third received normal hospital care. Functional capacity, muscle strength, cognition, mood, and QoL were compared. Gamification groups had greater improvements in Short Physical Performance Battery scores and Barthel Index scores (*p* < 0.05), but there were no significant differences between groups in cognition, QoL, or mood status (29).

This evidence on the impact of gamification on health outcomes measurement is mixed. The discrepancy may highlight artificial inflation on patient-reported outcomes (PROs) potentially attributable to activation of the body’s innate reward circuit from participating in game elements (35).

#### 3.1.5 Increased participant engagement

The published literature reported attempts to increase participant engagement in learning (1, 3), physical activity (17–19), symptom management (20), treatment (22), and follow-up care (21) via various gamification elements.

In the two studies targeting learning, both used cell phone applications to increase knowledge about the risks of burns in young children (3), and to increase adherence to therapeutic regimens in patients who had undergone coronary artery bypass graft surgery (1). In the first example, intervention participants used the “Cool Runnings” app to learn about burn risk and first aid via interactive quizzes and missions, and earned points for rewards, while the control group received only static infographics. In the second example, effects of educational app-based animated training were compared to in-person teach-back training on adherence to a therapeutic regimen.

Current literature suggested a minor improvement in patient engagement in psychopathological symptom management initiatives and physical activity in comparison to control interventions (17, 19). In one study, the use of gamification intervention was reported to increase physical activity in patients exposed to the intervention vs. those who were not (18). In a second study, Pham et al. assessed the efficacy of a cell phone application that aimed to improve anxiety, panic, and hyperventilation symptom management (20). Although patients found the application useful, no significant differences were found between the gamified and non-gamified approach in terms of psychopathology measures (i.e., anxiety assessed via the Generalized Anxiety Disorder Scale, panic assessed via the Panic Disorder Severity Scale-Self Report, and hyperventilation assessed via the Nijmegen questionnaire). In a third example targeting symptom management, Navarro-Alamán et al. described their methodology for developing and testing an app aimed at increasing patient engagement for the collection of PRO data from current or past patients with cancer, incorporating gamified elements like levels, points, progress display, feedback, and rewards, to enhance reinforcement, progress, and social connectivity (21). The authors reported that patient feedback was positive, and the application was easy to use while providing them with useful information around aspects of self-care such as relaxation. However, no impact on scores of how patients felt or functioned was reported.

A systematic review reported results of 10 clinical trials studies utilizing gamification (Nintendo Wii, VR, and computer games) for neurological motor rehabilitation (featuring conditions like cerebral palsy and developmental coordination disorder). These studies assessed outcomes (e.g., functional status, motivation, balance, strength, functionality, coordination, and satisfaction), assessed by COAs, which were improved in children and adolescents by the incorporation of gamification into treatment (22).

Finally, gamification has also been used to improve professional engagement in activities related to clinical trial conduct. Elements included tasks that were required to meet trial start-up timelines, which were gamified using a ‘Mount Everest climb’ to create friendly competition between sites. Scoreboards and site rankings were integrated into electronic trial management platforms, with progress shared in periodic webinars. Findings showed that increases in Mount Everest scores were associated with an increase in the probability of enjoying clinical trial sites during the start-up milestones earlier, with over half (52%–53%) of participants enjoying the game across two clinical trials, and 71% and 78% not feeling too much pressure from the rankings (6).

### 3.2 Surveys

Around a third of the US adults surveyed reported a lack of familiarity with augmented- or mixed-reality games (36%) and VR equipment and games (36%); clinical trial sites were often even less familiar with these (83% and 80%, respectively). Both adults and sites gave the highest preference to gamified mobile phone applications out of all gamification elements, resulting in a rank score of 3,526 for adults and 1,028 for trial sites. Traditional clinical trials without gamified elements were ranked as second preference by trial sites (rank score: 828) and third preference by US adults (rank score: 3,295) (Table 2).

**Table 2:** Summary of US adult and trial site survey results.

Question	US adults	Trial sites
How familiar are you with the following?	Those who are not familiar: 36% augmented reality or mixed reality games 36% virtual reality equipment and games 31% clinical trials 27% gamified mobile phone (cell phone) applications 19% computer games	Those who are not familiar: 83% augmented reality or mixed reality games 80% virtual reality equipment and games 74% computer games 51% gamified mobile phone (cell phone) applications
Would you prefer to take part in (manage) a clinical trial with the following?	Rank score: 1. A gamified mobile phone (cell phone) application (score: 3,526) 2. Computer game–based clinical trial (score: 3,391) 3. No gamified elements (traditional clinical trial) (score: 3,295) 4. Augmented or mixed reality game–based clinical trial (score: 2,785) 5. Virtual reality game–based clinical trial (score: 2,663)	Rank score: 1. A gamified mobile phone (cell phone) application (score: 1,028) 2. No gamified elements (traditional clinical trial) (score: 828) 3. Computer game–based clinical trial (score: 807) 4. Augmented or mixed reality game–based clinical trial (score: 711) 5. Virtual reality game–based clinical trial (score: 661)
How important would each of the following gamification elements be as part of a gamified clinical trial experience?	Rank score: 1. Customization (ability to choose) (score: 4,011) 2. Levels and progress feedback (score: 3,921) 3. Exploratory or open-world (score: 3,869) 4. Quests or challenges (score: 3,839) 5. Training and education (score: 3,784) 6. Point system or in-game currency (score: 3,761) 7. Use of videos (score: 3,759) 8. Storyline or narrative (score: 3,756) 9. Use of audio (score: 3,655) 10. Notifications (score: 3,575) 11. Mini games (score: 3,501) 12. Badges (score: 3,500) 13. Use of haptics (score: 3,309) 14. Social competition (score: 3,291) 15. Inclusion of mentors (score: 3,291) 16. Inclusion of peer groups (score: 3,272)	Rank score: 1. Notifications (score: 902) 2. Training and education (score: 859) 3. Use of videos (score: 854) 4. Use of audio (score: 830) 5. Levels and progress feedback (score: 813) 6. Customization (score: 789) 7. Inclusion of mentors (score: 780) 8. Storyline or narrative (score: 763) 9. Badges (score: 737) 10. Inclusion of peer groups (score: 724) 11. Quests or challenges (score: 724) 12. Personalization (score: 695) 13. Mini games (score: 687) 14. Exploratory or open-world (score: 686) 15. Social competition (score: 651) 16. Use of haptics (score: 641)
What do you think would be the main concern if gamification was used in a clinical trial?	Theme group: Human behavior and performance Reduced seriousness in relation to clinical trial (by participant) (*n* = 41) Loss of focus on trial (cause distraction) (*n* = 39) Concern over participants cheating or misusing the game (*n* = 38) Ability to understand games (including older participants) (*n* = 34) Gaming addiction (by participant) (*n* = 21) Concern over overly competitive or aggressive participants (*n* = 20) Demotivated (poor performers or rewards) (*n* = 8) Ethical concerns (being manipulated) (*n* = 6) Theme group: Data related Impact on data collected (bias, cheating) (*n* = 59) Data privacy (*n* = 49) Data security (including hacking) (*n* = 29) Level of accuracy required (*n* = 28) Data-sharing concerns (e.g., who, what, when) (*n* = 5)	Theme group: Impact on sites and patients More training required (patient and site) (*n* = 22) Increased site burden (*n* = 16) Increased costs (patient and site) (*n* = 14) Increased time burden (patient and site) (*n* = 14) Theme group: Human behavior and performance Older patients may have difficulties (*n* = 36) Patients’ ability to use the technology (physical, cognitive) (*n* = 13) Patients’ willingness to use the technology (*n* = 13) Learning curve may be too steep (*n* = 7) Theme group: Data related Data privacy (*n* = 18) Data security (*n* = 10) Data quality (*n* = 7)
	Theme group: Software (interface and game design) Account for learning curve and different physical and skill levels (novice gamers) (e.g., tutorials) (*n* = 30) Game design—games should result in satisfaction (*n* = 16) Level of gamification/immersion (concern too immersive) (*n* = 11) Game design—account for changes in skill levels during play (e.g., difficulty modes) (*n* = 8) Game design—provide good game graphics (*n* = 7) Game design—should be authentic (*n* = 6) Game design—provide control and customization (e.g., controls, sound, haptic feedback) (*n* = 6) Game design—don’t include micro-transactions (*n* = 5) Game design—provide different game genres/game types (*n* = 5) Game design—provide personalization (e.g., avatars) (*n* = 5) Game design—should be engaging and positive (*n* = 5)	Theme group: Practical Socio-economic differences (availability of technology or internet) (*n* = 12) Technical issues (including availability) (*n* = 9) Logistical issues (*n* = 8) Theme group: Sponsor concerns No added value/inappropriate for clinical trials (*n* = 8) Potentially reduced patient safety (*n* = 6)
	Theme group: Impact on clinical trials Time burden (for game completion) (*n* = 22) Maybe inappropriate for clinical trials (show compassion, credibility, clarity) (*n* = 20) Concern over increased complexity (*n* = 5)	
	Theme group: Practical Technological issues (e.g., technical faults) (*n* = 18) Access to required technology (*n* = 9) Costs incurred (*n* = 7)	
	Theme group: Health and safety Health impacts (general) (*n* = 12) Health impacts (cognitive) (*n* = 10) Safety (*n* = 10) Theme group: User experience Usability (accessibility, memorability) (*n* = 14) Variation in users over time (skills, dexterity, energy levels) (*n* = 11)	
What do you think would be the main benefit of gamification if used in a clinical trial?	Theme group: Improved clinical trial experience for patients Improved experience (*n* = 134) Reduced clinical trial burden (e.g., travel, flexibility, costs) (*n* = 16) Easier to use (*n* = 11) More inclusive and accessible (*n* = 6) More immersive (*n* = 5) Theme group: Improved patient engagement Improve engagement (*n* = 126) Improve adherence (*n* = 7) Provide competition and/or challenge (*n* = 6) Theme group: Improved recruitment Improve recruitment (*n* = 63) Appeal to younger generation (*n* = 19) New experience (*n* = 11) Theme group: Improved clinical trials for sponsors Better research results (*n* = 48) Ability to test patients’ skills, abilities, and behaviors (*n* = 14) More diversity (*n* = 9) Theme group: Improved educational experience Educational (*n* = 26) Theme group: Improved health and wellbeing Improved wellbeing (e.g., welcome distraction) (*n* = 24) Improved comfort (*n* = 6) Theme group: Improved social interactions Social interactions (*n* = 15) Help other patients (*n* = 9)	Theme group: Improved engagement Improved engagement (*n* = 54) Improved adherence/compliance (*n* = 23) More patient interactions (*n* = 6) Theme group: Improved recruitment Appeal to younger generation (*n* = 22) Improved recruitment (*n* = 5) Improved retention (*n* = 7) Theme group: Educational Educational (*n* = 18) Understandability (*n* = 8) Theme group: Improved patient experience More fun (*n* = 6) Easier for patients (*n* = 5) Theme group: Improved clinical trials Better research results (*n* = 7) Real-time feedback (*n* = 5)

*n* = number of comments. Themes with <5 comments have been removed.

Perceived concerns highlighted included the potential for reduced seriousness and focus on the trial, increased training and site burden, and the risk of participants cheating or misusing the game. Perceived benefits of gamification most frequently endorsed were improved recruitment, better research results, and enhanced patient experience through more engaging and fun trial processes (Table 2). The following sections provide a breakdown of survey responses.

#### 3.2.1 US adult surveys

In total, 1,044 adults responded to the survey, with enrollment achieving a balance across age groups. Each age group comprised 19%–20% of the sample [age (years) 18–24, *n* = 201, 19%; age 25–34, *n* = 209, 20%; age 35–44, *n* = 215, 21%; age 45–54, *n* = 212, 20%, and age ≥55, *n* = 206, 20%]. When asked whether they were living with illness, 59% of respondents classified themselves as healthy, 31% had mild or moderate illness, and 9% had serious illness; 1% were taking part in a clinical trial (Table 3).

**Figure 2:**
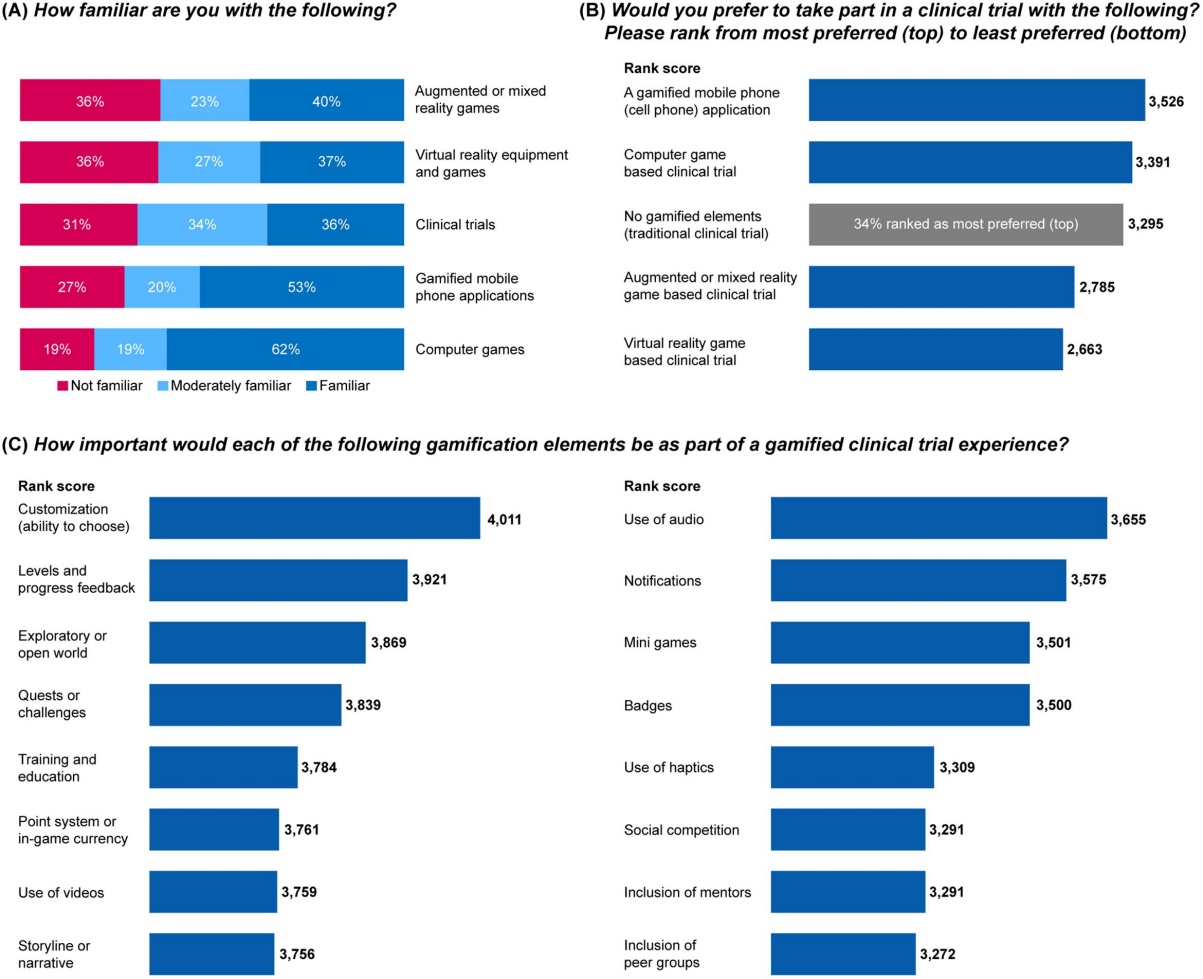
US adult survey findings. Familiarity **(A)** is represented as percentage of respondents to this question. To calculate the rank score **(B, C)**, each rank interval was assigned a number (e.g., 1 = not at all important, 5 = extremely important) with respondents choosing one rank per element. The number of respondents selecting each rank for each element was multiplied by the assigned number e.g., 1 × 16, then each was summed to provide the rank score. The total possible overall rank score was 5,220.

Respondents were most familiar with computer games (62%), followed by gamified cell phone applications (53%), augmented reality games (40%), and VR games (37%) (Figure 2A). Most would prefer to take part in a clinical trial that included a gamified cell phone application (rank score: 3,526) or a computer game–based clinical trial (rank score: 3,391) over either a traditional (rank score: 3,295) or a VR-based clinical trial (rank score: 2,663) (Figure 2B).

Respondents deemed the ability to customize the experience as the most important gamification element (rank score: 4,011), followed by levels and progress feedback (rank score: 3,921), an exploratory or open-world approach (e.g., options to allow respondents to choose their own path; rank score: 3,869), and quests or challenges (rank score: 3,839). The inclusion of training and education, points systems or in-game currency with goals, and storylines or narratives around the patient journey were deemed less important for a gamified clinical trial (Figure 2C).

Respondents thought that the use of other gamification elements such as audio (rank score: 3,655) and notifications (rank score: 3,575) were important, while haptics (technology that transmits tactile information using sensations such as vibrations, touch, and force feedback), social competitions (leaderboards), and inclusion of mentoring and peer groups (social networking) were considered to be less important (Figure 2C).

Low variability was observed in scores across the age groups; however, a clear pattern emerged when comparing the scores of the oldest age group (≥55 years) to the rest. Out of all age categories, those aged ≥55 years provided the lowest average rank score for all elements of gamification surveyed except notifications (e.g., reminders, texts), for which respondents aged 18–24 years provided the lowest rank score (3.34). Overall, average rank scores ranged from 2.46 to 4.03 across items. The highest averages were found in those aged 25–34 years, followed by 35–44 years then 18–24 years, across all elements of gamification surveyed. Specifically, these groups had the highest average scores for 10, four, and two out of 17 elements surveyed, respectively, with groups aged 18–24 years and 25–34 years ranking equal highest for ‘mini games to play along the way’ (Supplementary Table S3).

### Feedback on the US adult survey

“Use of challenges and quests to create a sense of adventure: This can help keep patients motivated and engaged in the trial process.” Male, aged 35–44 years

“Use of points, badges, and leader boards to track progress: This can help patients feel like they are making progress and that their participation is meaningful.” Male, aged 35–44 years

#### 3.2.1.1 Thematic and sentiment analyses

**Figure 3:**
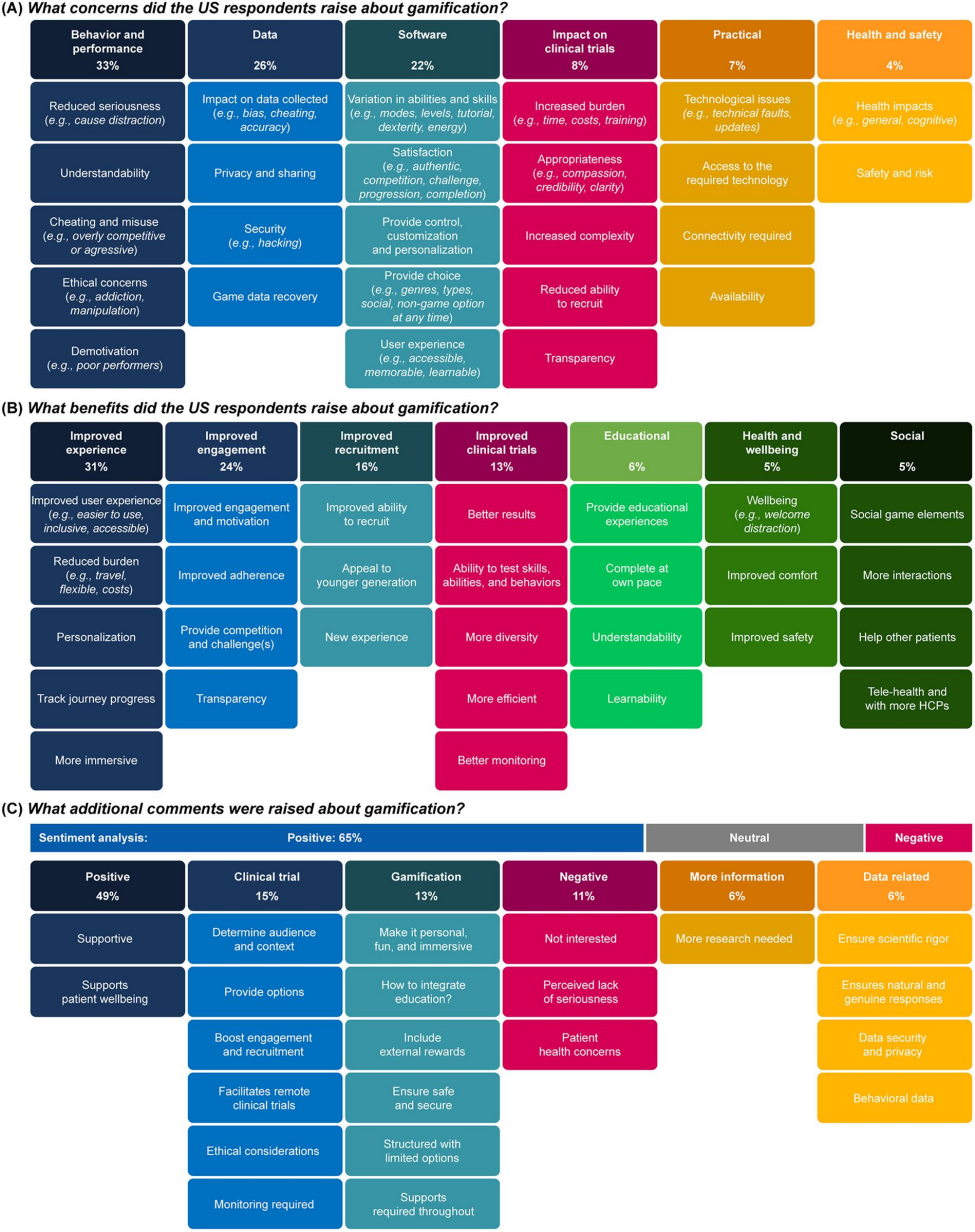
Thematic **(A,B)** and sentiment **(C)** analyses of US adult survey findings. For the thematic analysis, only open-text response options were requested, participant feedback was codified thematically. The frequency of participants from each adult perspective was quantified and bucketed into a matrix table of low, medium, and high, depending on the total frequency of participants within each code, and also grouped into a higher-order level theme. The sentiment analysis was conducted on additional comments raised about gamification whereby each adult respondent comment was grouped into positive, neutral, or negative overall. HCP, healthcare professional.

A thematic analysis of the adult respondents’ comments around their main concerns regarding gamification in clinical trials found that human behavior and performance (33% of comments), data security (e.g., hacking) and privacy (26%), and software (e.g., variation in abilities and skills, provision of choice: 22%) were the key topic areas, followed by impact on clinical trials (e.g., increased burden, increased complexity: 8%), practical concerns (e.g., technological issues, connectivity required and availability: 7%), and health and safety concerns (e.g., general and cognitive health impacts: 4%) (Figure 3A). Thematic analysis of the respondents’ comments around the potential benefits of gamification in clinical trials found the key topics were improvements in experience (e.g., easier to use and accessible: 31% of comments), engagement (e.g., improved engagement, motivation, and adherence: 24%), recruitment (16%), and clinical trials in general (13%) (Figure 3B). Sentiment analysis of the respondents’ comments about gamification in clinical trials found that 65% of comments were positive, 25% were neutral, and 10% were negative (Figure 3C).

#### 3.2.2 Clinical trial site surveys

Of the 311 sites surveyed (31% response rate), academic medical centers were the most common type of institution (44%). Most respondents were in managerial roles (90%); therapeutic areas were balanced between oncology (18%), dermatology (16%), and immunology, general medicine, and cardiology (15% each) (Table 3).

**Table 3:** Survey participant characteristics.

US adults (*N* = 1,044)	*n* (%)
Location	
USA	1,044 (100)
Birth sex	
Female	527 (50.5)
Male	516 (49.4)
Age, years	Age balanced [± 1%]
18–24	201 (19)
25–34	209 (20)
35–44	215 (21)
45–54	212 (20)
≥55	206 (20)
Rather not say	1 (0.1)
Currently living with an illness	
Healthy	616 (59)
Moderate illness	323 (31)
Serious illness	92 (9)
Taking part in a clinical trial	13 (1)
Clinical trial sites (*N* = 311)	
Institution	
Academic medical center	136 (44)
Private/community hospital	45 (14)
Dedicated research clinic	84 (27)
Private practice	44 (14)
Other	2 (0.6)
Experience	
Management	280 (90)
Research team	21 (7)
Specialist	10 (3)
Therapeutic area^a^	
Oncology	80 (18)
Dermatology	72 (16)
Immunology/inflammation	67 (15)
General medicine	66 (15)
Cardiology	65 (15)
Hematology	36 (8)
Rare and infectious diseases	32 (7)
Ophthalmology	23 (5)

^a^ Sites may cover more than one therapeutic area.

**Figure 4:**
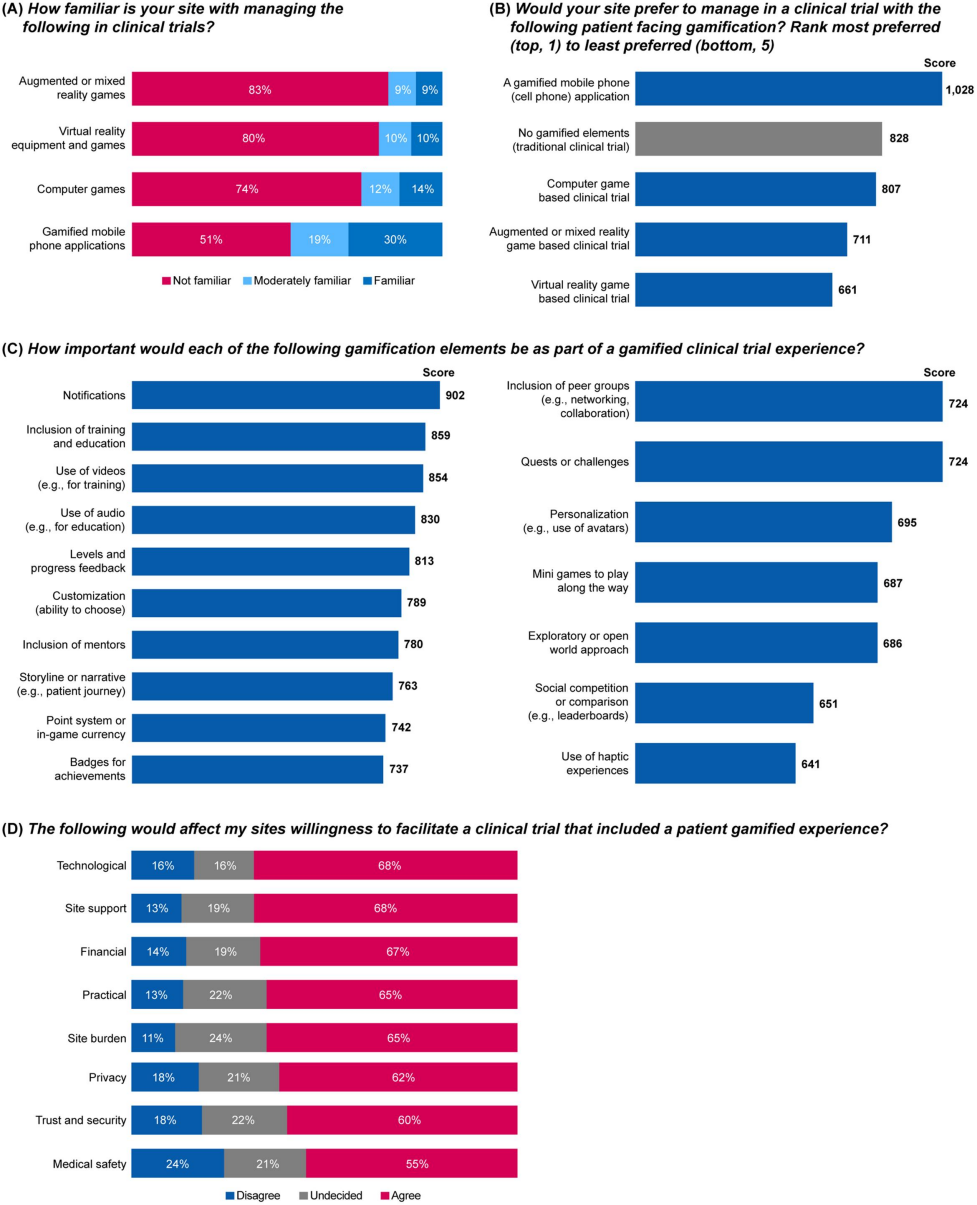
Clinical trial site survey findings. Familiarity **(A)** and agreement **(D)** are represented as percentages of respondents to each question. To calculate the rank score **(B, C)**, each rank interval was assigned a number (e.g., 1 = not at all important, 5 = extremely important) with respondents choosing one rank per element. The number of respondents selecting each rank for each element was multiplied by the assigned number e.g., 1 × 16, then each was summed to provide the rank score. The total possible overall rank score was 1,555.

Clinical study sites were most familiar with managing gamified cell phone applications (30%) in clinical trials, followed by computer games (14%), VR games (10%), and augmented reality games (9%) (Figure 4A). When asked about the types of clinical trials they would prefer to manage, most sites preferred to manage a clinical trial that included a gamified cell phone application (rank score: 1,028), followed by a traditional clinical trial (rank score: 828), and a computer game–based clinical trial (rank score: 807) (Figure 4B). Sites deemed the ability to provide notifications to their patients as the most important gamification element for a gamified clinical trial (rank score: 902), followed by training and education (rank score: 859), use of videos (e.g., for training; rank score: 854), and use of audio (e.g., for education; rank score: 830). Levels and progress feedback, customization, and inclusion of mentors were deemed less important by clinical trial sites (Figure 4C). Clinical trial sites rated social competition (rank score: 651) and the use of haptics (rank score: 641) as the least important gamification elements (Figure 4C).

Sites commented that ease of technological implementation (68%), impact on site support requirements (68%), and financial burden (67%) would affect their willingness to facilitate a clinical trial that included a gamified experience (Figure 4D).

The lowest average rank scores were reported for the academic medical centers site type across all except four elements of gamification surveyed [use of audio (e.g., for education), levels and progress feedback, inclusion of peer groups (e.g., social networking, collaboration with others), and social competition or comparison (e.g., leaderboards)]. Conversely, the private practice site type had the highest or equal average rank score for 12 of the 17 gamification elements surveyed, indicating a higher perceived importance of gamification from this site type compared to the others (Supplementary Table S4).

### Feedback from site survey

“Our trials recruit older or underserved populations without access to internet or devices. Some have no internet literacy. I’m not sure how we’d implement. Our staff are very good, but I have concerns about training and maintenance.” Academic medical center, Management

“I am highly concerned about these types of technologies worsening health disparities as [they] will need to be in multiple languages and [patients will need to] have access to tools.” Academic medical center, Management

#### 3.2.2.1 Thematic and sentiment analyses

**Figure 5:**
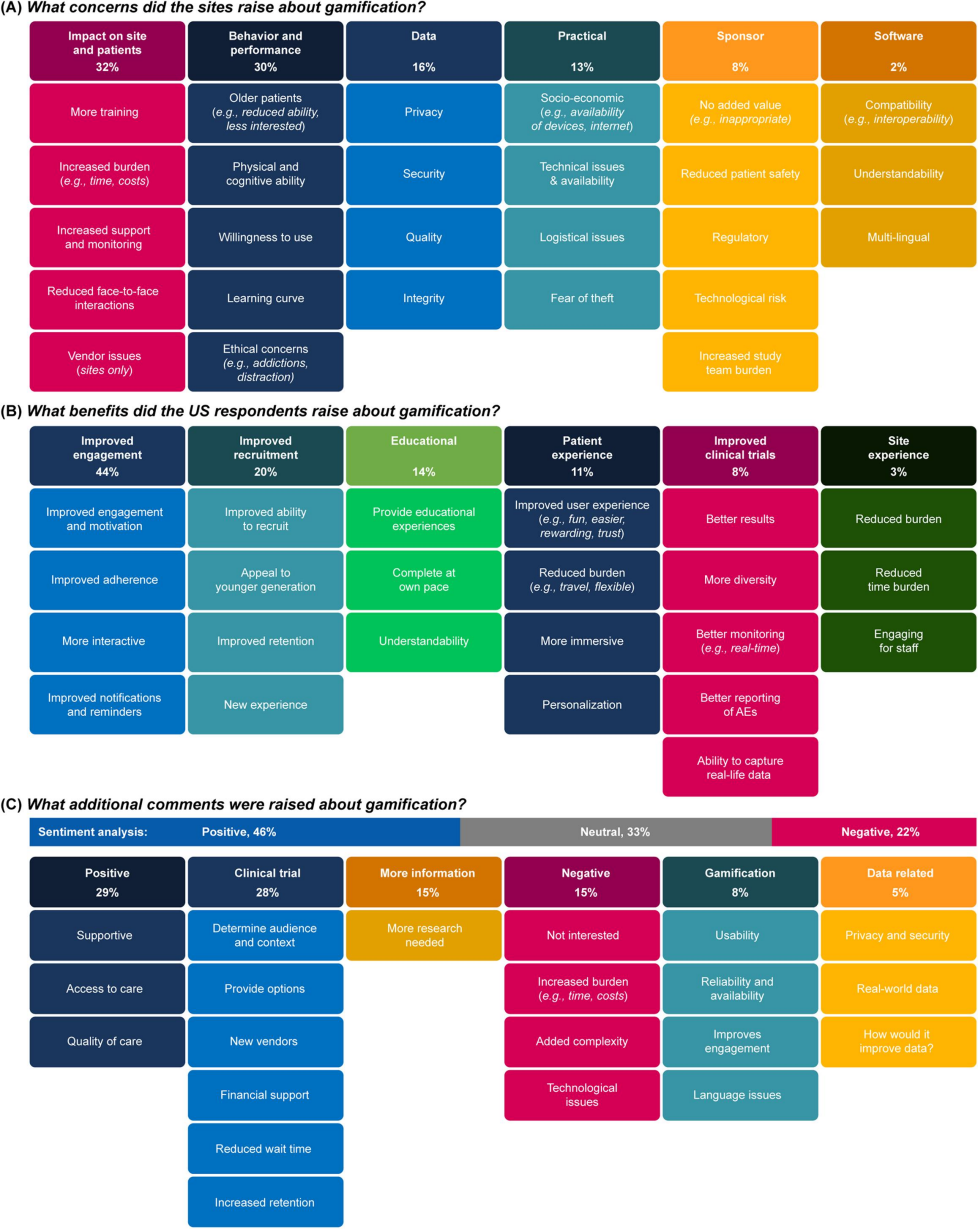
Thematic **(A,B)** and sentiment **(C)** analyses of clinical trial site survey findings. For the thematic analysis, only open-text response options were requested; participant feedback was codified thematically. The frequency of participants from each site perspective was quantified and bucketed into a matrix table of low, medium, and high, depending on the total frequency of participants within each code, and also grouped into a higher-order level theme. The sentiment analysis was conducted on additional comments raised about gamification whereby each site respondent comment was grouped into positive, neutral, or negative overall. AE, adverse event.

Thematic analysis of the sites’ comments found that the key topic areas of concern were impact on site performance (32% of comments) and human behavior and performance (e.g., older patients, physical and cognitive ability, willingness to use: 30%); data concerns were deemed less important (e.g., privacy, security, and quality: 16%), followed by practical concerns (e.g., availability of devices and internet, technical issues: 13%), impact on the sponsor (e.g., reduced patient safety, regulatory: 8%), and software concerns (e.g., compatibility and interoperability, understandability: 2%) (Figure 5A). Thematic analysis of the sites’ comments around the potential benefits of gamification in clinical trials revealed that improvements in engagement (44% of comments), recruitment (20%), and education (e.g., provision of educational experiences and the ability to complete at own pace: 14%), as well as patient experiences (e.g., fun and rewarding, reduced travel burden: 11%) were considered key benefits (Figure 5B). Finally, in a sentiment analysis of the sites’ additional comments around the topic of gamification in clinical trials, 46% of comments were positive, 33% were neutral, and 22% were negative (Figure 5C). Of note, sites appeared to be less positive about the inclusion of a gamified experience in clinical trials than were the adult respondents (Figure 3C).

## 4 Discussion

This study describes current literature investigating gamification in clinical trials, and current perceptions on gamification held by US adults and clinical trial sites. Current literature review findings highlight that previous research generally resulted in mixed evidence of the advantages of gamified interventions, with about half of the studies suggesting a positive impact on health outcomes and patient engagement (1, 3, 6, 18, 21, 22, 29).

This review reports articles exploring gamification aimed at enhancing clinical research participation, health outcomes measurement, and participant engagement. The Paper Kingdom Game increased children’s knowledge about clinical trials but was less effective than traditional methods. Exergames like Microsoft Kinect showed mixed results: standard physiotherapy outperformed gamified methods for shoulder impingement syndrome, while hospitalized older adults saw improved physical performance but no cognitive or QoL benefits. Gamification increased knowledge about burn risks and post-surgery regimen adherence but showed limited benefits in psychopathological symptom management and physical activity. Positive outcomes included improved engagement in collecting PROs for cancer survivors and accelerated clinical trial start-up activities. Key gamification elements included notifications, rewards, multi-modal interactions, and education, but potential risks like competition-induced demotivation, notification fatigue, and technological limitations must be managed.

Hamari et al. previously described that, while many articles report positive findings, the majority conclude positive effects in only part of the results (7). The current review also identified mixed findings for and against the advantages of gamification within studies. Many studies lacking adequate designs were identified—such as small sample sizes, lack of instruments that are fit for purpose, lack of control groups, and short follow-up periods, which may hinder an accurate assessment of gamification’s true impact.

Another possible application of gamification in clinical trials is to capture PRO data, the importance of which has been highlighted by bodies such as the US Food and Drug Administration (36). Literature review results highlighted the limited focus in the existing literature on how gamification impacts the assessment of how patients feel and function, as measured by different types of COAs in a clinical trial. Still, no evidence of gamification being used to collect PRO data has been found (e.g., a gamified version of a daily diary or gamified handheld device to encourage data completion).

In the second part of our study, over 1,000 US adults and 300 global clinical trial sites were surveyed on their perceptions and acceptance of gamification in clinical trials. Our findings indicate that, while most respondents had familiarity with gamified cell phone applications, clinical trial sites were slightly less familiar with managing gamification in a clinical trial context. Sites noted concerns about patients having limited access to internet/devices and low internet literacy. However, both groups said they would prefer gamified cell phone applications over a traditional approach within clinical trials. US adults and clinical trial sites had different priorities when it came to gamified elements. Training and education were identified as the most important elements for sites, while patients prioritized customization and levels/progress on feedback.

The survey results provide useful insights for consideration in the design of future gamification elements in clinical trials. Although current findings suggest that certain elements of gamification are viewed positively from both patients’ and sites’ experiences of clinical trials, implementation must be done thoughtfully to enhance the likelihood of increasing engagement including consideration of user preferences, context, motivational affordances, and user characteristics. This should not only take user preferences into account, but also precise and reliable data on whether gamified elements would create a positive impact, as well as tailoring to the specific context of use.

In our study, older participants had the lowest average rank scores for all except one gamification element, showing the lowest perceived importance across gamification elements except for notifications. This was unfavorable to the lowest age group (18–24 years), which may indicate low tolerance for additional notifications in this age range. This may be due to a lack of familiarity or comfort among the elderly given that the advent of gamification is fairly recent. A less clear pattern was found in the sites surveyed, where private practice appeared to perceive the gamification elements as more important while academic medical centers perceived many of the same elements as less important. Prior studies have identified facilitators and barriers associated with the use of technology in clinical research, particularly among certain populations having issues with technology access and/or usability. For example, older adults may not have the same level of familiarity with technology compared to younger populations; very young individuals or individuals with cognitive or functional impairments may have more difficulty using technology; and individuals with low income may lack access to reliable internet connections or the technology required for gamification to be enabled. Findings from the current study may inform future targeted follow-up surveys to further investigate elements such as differing attitudes between different countries or patient populations, and/or conduct further investigation into differences among relevant demographic or clinical subgroups (e.g., those disadvantaged by a lack of digital health equity, or differently abled participants).

Implementation of gamified approaches may have a higher probability of success when relevant factors are considered. These approaches may include audience and context, variation in participant abilities, skills, and energy levels and impact on the user experience (e.g., burden, complexity, distraction, misuse, ethical, competition, rewards, and demotivation). In addition, data requirements (e.g., privacy, security, and recovery), integration with other clinical trial technologies and tasks, and maximizing user satisfaction [e.g., social, an authentic experience (defined as an experience that is emergent, unscripted, and unique, and when reflected upon serves as a learning tool for adults) (37), challenge, progression, and completion] are other gamified approaches to be considered. Implementation of these can be achieved by offering choice, control, customization, and personalization, providing access to and addressing potential issues of the technology (e.g., connectivity and type required), and providing support, training, and educational experiences.

These findings may also encourage the investigation of simplified, less technical alternatives for gamification elements that could mitigate issues with access or usability experienced by different age groups. For example, to ensure equitable access and minimize the risks due to widening health disparities, practical mitigation strategies should be considered when implementing gamification in clinical trials, such as:

- Use of iconography or audio instructions to reduce reliance on text interfaces for low-literacy participants.
- SMS-based reminders, paper-based gamified diaries, and telephone-based engagement for participants with technological limitations (e.g., no smartphones or internet access).
- Options for choosing interfaces in multiple languages, including culturally adapted content to improve comprehension and engagement.
- Options for increasing font sizes or simplified navigation for older adults or those with ability limitations (e.g., vision impairment, cognitive or physical impacts).
- Designing apps that work without continuous internet connectivity and synchronize data when a connection becomes available.

In both the US adult and clinical trial settings, this study only surveyed participants within the US. Responses from the US adult survey were gender- and age-balanced across groups but there were no additional restrictions used to analyze the data. Clinical surveys were conducted at 1,000 US sites; these sites were identified from an internal database and thus had previously engaged with the study sponsor. These limitations should not limit the findings due to the large number of participants included in the surveys.

Based on the results presented here, gamification requires further exploration and validation for its use in the context of clinical trials. Although not found within the results of the current study, there is a potential impact of gamification whereby scores on PROs may be artificially inflated due to activation of the body's innate reward circuit (38). Further studies are needed, especially to understand potential impacts on how patients feel and function, potential interference with demonstration of treatment benefit, and to inform judicious future use. Continued feedback from users is critical to allow adjustment of gamified approaches to fit users’ needs and ensure that risks are mitigated. Finally, use of gamification should acknowledge and balance the inherent risks and potential value.

Conclusions from these findings must be made in the context of the study's limitations. The purpose of this review was not to provide a systematic review and synthesis of gamification across all healthcare contexts, but rather to focus comprehensively on practical applications within clinical trials and inform trial design and innovation. Our focused approach evaluating the application of gamification specifically to clinical trials was designed to inform a context bordered by unique regulatory, methodological, and operational considerations, which adds practical relevance for trialists and other stakeholders. While our review provides a focused perspective on the application of gamification within clinical trials, we acknowledge this scope limitation may underestimate the breadth of existing evidence, such as compared with a review by Salehi et al. (39), which systematically examined gamification in medical education contexts. Future research should identify broader themes by reviewing broader applications of gamification and should enhance transparency and interpretability by incorporating a more formal grading system to evaluate the risk of bias and methodological rigor in a systematic manner.

Survey respondents comprised a convenience sample of US adults from an online database, which may not be a fully representative sample of the US population and may limit the generalizability of current findings. Further data are needed to ascertain whether this impacts findings.

In conclusion, gamification presents a significant opportunity to increase patient engagement and capture PROs effectively. Pharmaceutical sponsors may benefit from a balanced implementation of gamification, taking into account the context of use and target population. By addressing the identified factors and limitations, gamification may be a valuable tool in enhancing clinical trial processes and outcomes.

## Data Availability

The datasets presented in this article are not readily available because qualified researchers may request access to study documents that support the methods and findings reported in this manuscript. Individual anonymized participant data will be considered for sharing if there is legal authority to share the data and there is not a reasonable likelihood of participant re-identification. Requests to access the datasets should be directed to https://vivli.org/.
