# Supplementary material for: Practical applications of gamification in patient-centered outcomes research and digital health, and its acceptance in clinical trials
Source: Front Digit Health. 2026 May 29;8:1652217. doi: 10.3389/fdgth.2026.1652217 (PMC13260181; doi:10.3389/fdgth.2026.1652217)
Supplement: Supplementary file 1 [file Table1.docx]

# Supplementary Table 1. Survey questions

| **US adult questions** | **Clinical trial site questions** | **Options** |
| --- | --- | --- |
| How familiar are you with the following?  (not familiar, moderately familiar, familiar) | How familiar is your site with managing the following in clinical trials?  (not familiar, moderately familiar, familiar) | Augmented or mixed reality games  Virtual reality equipment and games  Clinical trials  Gamified cell phone applications  Computer games |
| Would you prefer to take part in a clinical trial with the following? Please rank from most preferred (top) to least preferred (bottom) | Would your site prefer to manage a clinical trial with the following patient facing gamification? | A gamified cell phone application  No gamified elements (traditional clinical trial)  Computer game-based clinical trial  Augmented or mixed reality game-based clinical trial  Virtuality reality game-based clinical trial |
| How important would each of the following gamification elements be as part of a gamified clinical trial experience? | How important would each of the following gamification elements be as part of a patient gamified clinical trial experience? | Badges  Customization  Exploratory or open world  Inclusion of mentors  Inclusion of peer groups  Levels and progress feedback  Mini games  Notifications  Personalization  Point system or in-game currency  Quests or challenges  Social competition  Storyline or narrative  Training and education  Use of audio  Use of haptics  Use of videos |
|  | The following would affect my sites willingness to facilitate a clinical trial that included a patient gamified experience…  (agree, disagree, undecided) | Technological  Site support  Financial  Practical  Site burden  Privacy  Trust and security  Medical safety |
