# Supplementary material for: Practical applications of gamification in patient-centered outcomes research and digital health, and its acceptance in clinical trials
Source: Front Digit Health. 2026 May 29;8:1652217. doi: 10.3389/fdgth.2026.1652217 (PMC13260181; doi:10.3389/fdgth.2026.1652217)
Supplement: Supplementary file 2 [file Table2.docx]

Supplementary materials

Supplementary Table 2. Summary of articles utilizing gamification

| **Study** | **Area of application** | **Objective** | **Design** | **Gamification definition** | **Gamification element** | **Results: Impact on trial engagement/performance and/or patient outcomes** | **Was evidence positive/in support of gamification?** |
| --- | --- | --- | --- | --- | --- | --- | --- |
| **Marceau et al. unpublished (35)** | **Education about clinical trials** | Gamified education to inform children aged 8–14 years about their rights in participation in clinical trials, and reduce their fears about participation | Prospective controlled study comparing knowledge between children who learned from traditional (paper-based) versus gamified trial educational material | Not provided | Role-playing game simulation | Paper-based learning group showed superior outcomes (greater knowledge about clinical trials) | X |
| **Nazmi et al. (15)** |  | To evaluate the efficacy of gamification in education of teenage females on pubertal health, specifically assessing changes in puberty awareness and practice among adolescent girls | Randomized controlled trial conducted in middle schools for girls in Babol, Iran, with multistage cluster sampling, randomization, parallel design, no reported blinding, intervention (gamified education) versus control (standard education), duration 4 weeks | Gamification involves incorporating game mechanics, techniques, and elements into educational and nongame settings to motivate and engage participants in reaching specific learning, professional, and health objectives | Points, leaderboards, badges, levels, challenges (multiple-choice, descriptive, key point reviews), rewards | Significant improvement in both awareness and practice in the intervention group versus control, effect sizes 0.74 (awareness) and 0.25 (practice), *p* < .001. Participant feedback was positive; gamification was described as engaging and enjoyable | ✓ |
| **Ghafouri et al. (16)** |  | To compare the effect of education using the flipped class, gamification, and gamification in the flipped learning environment on the performance of nursing students in a client health assessment | Parallel randomized clinical trial with 166 nursing students in a university setting, randomized into four groups (gamification in flipped learning, gamification, flipped class, lecture); intervention lasted 4 weeks | Includes the use of game elements to increase motivation and participation and to involve students in the personal learning process | Points (green/red/yellow carts), scenario-based challenges, competition, rewards, narratives, user-friendly aesthetics | Significant differences in knowledge and satisfaction scores favoring gamification in a flipped learning environment (*p* < .05). No significant difference in self-efficacy. Gamification methods were associated with higher satisfaction and attractiveness | ✓ |
| **Burgess et al. (3)** | **Increasing participant engagement** | Investigate the impact of a gamified version of the ‘Cool Runnings’ app on knowledge about risk of burns and first aid for burns | Two-group randomized controlled trial including an intervention group that utilized a gamified app interface and a control group that utilized an app interface that did not incorporate gamified techniques | Gaming principles of rewards, competition, and personalization to engage participants and motivate them toward preferred behaviors | Gamified app interface incorporating pop quizzes, ‘missions’ (e.g., photo uploads) that reinforced intervention message themes, points-based competition, and rewards | Intervention (i.e., gamification) group showed significantly greater improvement in overall knowledge about burns and greater engagement with the app compared to the control group | ✓ |
| **Ghorbani et al. (1)** |  | Compare the effects of gamification and teach-back training methods on adherence to a therapeutic regimen in patients after coronary artery bypass graft surgery | Randomized clinical trial comparing two experimental intervention groups (gamification and teach-back) and a control group (usual care or routine training) | Concept of applying game design and mechanics to non-gaming applications | Gamification app incorporating animated training on dietary regimen, medication regimen, and physical activity regimen, as well as points-based competition | The gamification training method performed significantly better than the teach-back training method and routine training in increasing dietary and movement regimen adherence. There was no statistically significant difference in adherence to medication regimens between the two groups | ✓ ^a^ |
| **Edney et al. (17)** |  | Investigate the impact of a social and gamified app on physical activity levels | Three-group cluster randomized controlled trial including two experimental intervention groups (gamified app and basic app) and a control group (no gamified intervention) | Integration of game-like elements into non-game environments | Gamification app (Active Team) encouraging 10,000 steps per day for 100 days, incorporating daily step count goals and self-monitoring of goals, as well as social and gamified elements (e.g., connecting existing friends via Facebook) mimicking real-life social interactions | No significant differences were found between groups in objective physical activity; however, the gamified intervention was associated with significantly greater levels of self-reported moderate-to-vigorous physical activity. No significant differences were observed between groups for outcomes of quality of life, depression, anxiety, stress, and wellbeing | X |
| **Patel et al. (18)** |  | Examine the effectiveness of gamification interventions to increase physical activity among overweight and obese adults | Randomized clinical trial comparing three experimental gamification intervention groups (support, collaboration, and competition) and a control group (usual care or routine training) | The use of game design elements, such as points and levels, in non-game contexts | Gamification group: points and levels for achieving step goals. Support gamification: encouragement and motivation from a selected family member or friend. Collaboration gamification: Assignment to a team of three in which one participant was randomly selected each day to represent the team, and the team would win or lose points based on the representative's achievement of their daily goal. Competition gamification: Assignment to a team of three in which a leaderboard was used to rank participants and encourage them to compete for the top spot | All gamification groups showed significantly greater increases in mean daily steps over 9 months in comparison to the control group. Participants in the gamification group based on competition showed the greatest increase; this was the only group in which physical activity remained significantly greater than that of the control group over a 12-week follow-up period | ✓ |
| **Kurtzman et al (19)** |  | Examine the effectiveness of gamification interventions for the promotion of weight loss | Randomized controlled trial including two intervention groups (gamification and gamification with primary care physician input) and a control group (no gamified intervention) | The use of game design elements, such as points and levels | Gamification group participants entered a points-based game in which they gained or lost points based on the achievement of weekly weight-loss targets. One of the gamification arms had weight and step data shared regularly with each participant’s primary care physician | All groups showed significant weight loss over the course of the trials; however, there were no significant differences in weight loss between the gamification and control arms | X |
| **Pham et al. (20)** |  | Evaluate the feasibility and clinical efficacy of a mobile health game (Flowy; www.flowygame.com) | Web-based, parallel-group randomized controlled trial involving an intervention (Flowy) group and a waitlist control group | The implementation of the most common and enjoyable mechanics of videogames in non-videogame contexts | Minigames which require players to use breathing retraining exercises and perform diaphragmatic breathing to play/progress in order to alleviate anxiety and panic, as well as manage hyperventilation symptoms | Participants in both the intervention and control groups reported improved scores in anxiety (Generalized Anxiety Disorder Scale-7 item; Overall Anxiety Severity and Impairment Scale and Anxiety Severity Index-3 item), panic (Panic Disorder Severity Scale-Self Report), hyperventilation (Nijmegen Questionnaire), and quality of life (Quality of Life Enjoyment and Satisfaction Questionnaire-Short Form). However, a group-effects multivariate analysis of variance showed no significant difference for psychopathology measures (anxiety, panic, and hyperventilation) based on treatment condition | X |
| **Navarro-Alamán et al. (21)** |  | Develop and test a gamification app for people who have survived cancer aimed at increasing patient engagement when collecting patient-reported outcome data | Development and application of a methodology for the creation of gamified health apps, and prospective testing of the feasibility and acceptability of an app designed based on this methodology | The use of game mechanics in non-play environments and applications in order to enhance motivation, concentration, effort, and loyalty | The app incorporated three gamification strategies (reinforcement, progress and social connectivity), and five gamification tactics (using levels, allocating points, showing progress, providing feedback, and giving rewards) | Descriptive statistics showed that participants considered the app easy to use and that it provided useful information | ✓ |
| **Pimentel-Ponce et al. (22)** |  | Review clinical trials using gamification in rehabilitation treatments in children and adolescents with neuromotor impairment | Systematic review | The use of game-related elements in a non-play setting e.g., workplace, education, or healthcare contexts | Study-specific; exemplified games (e.g., Nintendo Wii Fit training) included elements such as points, levels, and showing progress | The review showed that outcomes in motivation, balance, strength, functionality, coordination, and satisfaction, among others, were improved by incorporating gamification into the treatment of neuromotor conditions in children and adolescents | ✓ |
| **Lane et al. (6)** |  | Explore the effects of gamification to improve the conduct of clinical trial activities | Gamification of clinical trial platform tasks and retrospective evaluation of the impact of gamification on task achievement | The application of typical elements of game playing (e.g., point scoring, competition with others, and rules of play) to other areas of activity like the workplace or (in the present case) clinical trials, to produce the desired effects | Gamification was applied by simulating a Mount Everest climb in which reaching the summit equated to trial activation. Start-up tasks were assigned point values and bonuses/penalties were received for early/late completion. Scoreboards were built into electronic trial management platforms; trial and game information (including scores and site rankings) was shared in webinars in which recognition was provided for accomplishments | A higher Mount Everest score was significantly associated with an increase in the probability of achieving start-up milestones more quickly. The probability of completing site activation faster was increased by 61% for every 32 points in the Mount Everest score. Game enjoyment appeared to moderate performance; those who enjoyed playing the game, on average, had higher Mount Everest scores | ✓ |
| **Xu et al. (23)** |  | The purpose was to examine the impact of a smartphone-based gamification intervention on physical activity participation and various relevant physical and psychological outcomes in coronary heart disease patients | Single-blind, randomized controlled trial with three arms (control, individual, team) at a hospital in Changchun, China. 108 participants were randomized; interventions lasted 12 weeks with 12-week follow-up. The control group received step goal setting only; individual and team groups received gamified behavioral intervention via WeChat applet. Randomization was parallel; blinding was single-blind. Duration of intervention was 12 weeks | Gamification is the use of game design features (such as points, leaderboards, and progress bars) to improve motivation and engagement | Points, levels (bronze, silver, gold, platinum, diamond), rewards, progress-based feedback, teams (social interaction), step goals, leaderboards | For the individual group, gamification significantly increased PA (step count difference 988; 95% CI 259–1717; *p* < .01) and maintained effect during follow-up. Significant improvements in competence, autonomous motivation, body mass index, and waist circumference were observed. Team group showed increased competence, relatedness, and motivation but no significant physical activity increase. Qualitative feedback: “The game is useful and exciting, I want to do walk more to keep my points.” Some team members felt frustrated by lack of contact with teammates. Overall, gamification had a positive impact on physical activity and motivation, with mixed effects for team-based gamification | ✓ |
| **Gavish et al. (24)** |  | To verify whether neck movements invoked by a fully immersive virtual reality (VR) game environment may be considered comparable with physiotherapist-prescribed rehabilitation exercise | Single-visit, prospective clinical trial (NCT03104647) at a medical center with 20 healthy participants. All performed a VR-based gamified neck movement session; movements recorded and assessed by physiotherapists. No randomization or blinding; all participants received the intervention. Duration: one session | By providing immediate feedback and gamification, the VR environment enhances motor learning and delays the onset of boredom from the therapeutic exercises, thus improving patient adherence to the rehabilitation program | Immediate feedback, immersive VR environment, game-based tasks (filling barrels), real-time performance feedback. | All 320 movements (16 per participant) were identified as appropriate for rehabilitation by physiotherapists. No adverse events. Authors note potential advantage of home-based VR gamification to motivate adherence: “The potential advantage of home-based VR gamification of cervical spine rehabilitation programs over common practice in motivating patient adherence warrants evaluation by randomized controlled trials.” | ✓ |
| **Imran Ho et al.** **(25)** |  | The primary objective was to identify and assess game elements incorporated into smoking cessation applications and evaluate the effectiveness of gamified interventions on smoking cessation outcomes. Secondary objectives included synthesizing current evidence on gamification’s impact, identifying behavioral change mechanisms, and providing actionable insights for mHealth tool design | Systematic review and meta-analysis of 15 randomized controlled trials with 5,075 participants. Studies compared gamification-based smoking cessation strategies with non-gamified controls. Most interventions were delivered via mHealth apps, with parallel group design and intervention durations ranging from 3 to 12 months. Outcomes were narratively synthesized and pooled by follow-up time. Randomization was present; blinding status varied | The use of game design elements in non-game contexts | Competition, milestone recognition, storytelling, rewards (points, badges, tokens), leaderboards, progress tracking, achievement badges, goal setting, progress tracking, levels, social sharing, narrative, adaptive feedback | Gamification-based interventions significantly improved smoking abstinence rates compared to non-gamified controls (relative risk=1.91 for <6 months, relative risk=1.37 for ≥6 months, both *p* < .05). Positive participant feedback highlighted increased motivation, engagement, and self-efficacy, especially in short-term outcomes. Long-term effects were attenuated but remained significant. “Integrating gamification elements leads to markedly higher smoking abstinence rates compared to non-gamified interventions.” Limitations included inability to isolate individual element effects and heterogeneity in outcome measures | ✓ |
| **Litvin et al. (26)** |  | The primary research objective was to investigate the effect of a gamified mobile mental health app (eQuoo) on resilience and mental health in university students, comparing outcomes to a non-gamified app and a waitlist control. Secondary objectives included examining attrition rates and engagement, and assessing the utility of gamification in digital mental health interventions | Large-scale randomized controlled trial with three arms: gamified app (eQuoo), non-gamified app (Sanvello), and waitlist control. Participants were university students (≥18 years) recruited online. Parallel group design; intervention duration not specified. Randomization present; blinding status not specified | One approach to combating attrition and nonengagement in digital health interventions design is the use of game design elements in nongaming contexts or ‘gamification’. Of the 18 gamification elements...11 have been incorporated into eQuoo: levels, points, rewards, narratives, personalization, customization, mini games, quests and challenges, badges, artificial assistance, unlockable content | Levels, points (gem shards), rewards (unlocking levels/gems), narratives, personalization (story choices), customization (avatar), mini games, quests/challenges (stories), badges (personality types), artificial assistance (guide), unlockable content | The gamified app significantly improved resilience and mental health outcomes compared to both control groups. Attrition rates were lower in the gamified group. “This study supports...benefits of digital health interventions incorporating gamification and resilience training...mobile mental health apps can assist in mental health treatment and resilience building.” Participant feedback indicated increased engagement and enjoyment. Limitations included reliance on self-report and inability to isolate which gamification elements were most effective | ✓ |
| **Greysen et al (27)** |  | To test the effect of a behaviorally designed gamification intervention with a support partner on physical activity in older adults at risk for Alzheimer's disease and related dementias | Randomized controlled trial, national registry recruitment, adults aged 55–75, intervention and control groups, remote delivery, 12-week intervention with 3-week follow-up, not blinded to participants but blinded analysis | The application of game design elements in non-game contexts | Points, levels (blue, bronze, silver, gold, platinum), precommitment pledges, social support partner, progress feedback, status, loss aversion | Intervention group increased mean daily steps by 1,699 steps/day (*p* < .0001) and sustained gains in follow-up. Increased moderate-to-vigorous physical activity. Gamification with social support was effective in promoting and sustaining higher physical activity | ✓ |
| **Barratt et al. (28)** | **Measurement of health outcomes** | Investigate use of exergames for patients with shoulder impingement syndrome who have undergone arthroscopic subacromial decompression | Randomized prospective controlled trial comparing the effects of physiotherapy aided by automated sensor-based technology for performing exergames and standard physiotherapy | Use of game design elements in a non-game context to improve user experience and engagement | Exergame-enabled physiotherapy | Significant increase (improvement) observed for the control group at post-operative assessment while the exergames group showed no significant change in clinical outcome assessments (Oxford Shoulder Score, Disability of Arm, Shoulder, and Hand tool scores). No significant changes seen in either group in EuroQoL 5-Dimension questionnaire scores | X |
| **Cuevas-Lara et al. (29)** |  | Investigate the effect of gamified interventions on functional capacity in hospitalized older adults | Non-randomized controlled trial comparing two experimental intervention groups (simple gamification and technology-based gamification) and a control group (usual hospital care, not including physical rehabilitation) | Full games or game elements whose final goal is not the training itself but the promotion of changes in the use or acquisition of new knowledge, in attitudes, or in physical, cognitive and/or social abilities | Simple gamification: gamified activity diary. Technology-based gamification: the Health Arcade prototype (includes movement detection sensors, wireless communication systems, feedback systems [e.g., screens], and virtual reality) | Gamification groups showed greater improvements in Short Physical Performance Battery scores and Barthel Index scores in comparison to the control group. No significant differences were observed between the intervention groups and control groups for cognition, quality of life, or mood status | ✓ |
| **Gillcrist et al. (30)** |  | To evaluate a behaviorally designed intervention utilizing gamification and social support to improve physical activity and reduce symptoms in patients with knee osteoarthritis (KOA). | Double-blind, factorial randomized controlled trial at a VA Medical Center, enrolling veterans with KOA. Participants received a Fitbit and completed a 2–4 week baseline period. Intervention lasted 32 weeks. Randomized to intervention (gamification + social support) or control (weekly updates only). Parallel design; randomization and blinding described | Not provided | Points, levels, medals, social support partner, weekly feedback, step goals | Intervention group walked 1,119 more steps per day (95% CI –562, 2,799; *p* = .19) versus control. Greatest effect in first 6 months. Knee outcome improved over time in intervention group (mean 2-week change +0.62 versus –0.38; *p* = 0.02). Qualitative: “Interventions combining social support and gamification elements can provide benefit to individuals with KOA...” No statistically significant differences in primary outcome, but trends favored intervention | X/✓ |
| **Weller et al. (31)** |  | To test feasibility and efficacy of a gamified app-based cognitive control training (de:)press ©) for reducing depression severity compared to a non-gamified version. | Randomized controlled pilot trial with two parallel active groups (gamified intervention versus non-gamified control), adult patients with major depressive disorder, 6-week intervention with three sessions/week, followed by 4-week discretionary use; no blinding reported | The use of gaming elements in non-game contexts | Purpose-driven narrative, feedback (immediate and long-term), ownership, challenge, reward, progression graphs, achievements, avatars, levels | Greater decrease in depression severity (Montgomery-Åsberg Depression Rating Scale scores) in gamified group at 4-week follow-up, improved training adherence, positive usability ratings, no severe adverse effects. Impact described as positive and sustainable for depressive symptoms | ✓ |
| **Wang et al. (32)** |  | To summarize randomized clinical trials investigating the effects of serious games (including gamification) on cognitive and functional outcomes in patients with mild cognitive impairment | Narrative review of eight randomized control trials: designs included single-blind, double-blind, and open-label; settings varied (hospital, community, supervised training, VR/online platforms); interventions included cognitive training games, exergames, and combinations; durations ranged from 4 to 18 weeks | Serious games are defined as games primarily designed for learning and education, rather than entertainment, making them an exciting avenue for cognitive intervention. Gamification is discussed as the integration of game elements into therapeutic digital interventions. | Computerized cognitive training games, VR-based exergames, avatars, progression tracking, personalized game elements, feedback, rewards, physical challenges | Across studies, serious games and gamification led to improved cognitive function, quality of life, motivation, enjoyment, and physical abilities. Personalized game elements enhanced outcomes. All randomized control trials reported positive or superior efficacy compared to conventional therapy or usual care | ✓ |
| **Jiang et al. (33)** |  | The primary aim was to develop and evaluate a remote pulmonary rehabilitation (PR) intervention based on gamification and Health Action Process Approach (HAPA) theory for older adults with chronic obstructive pulmonary disease (COPD). Secondary aims included exploring mechanisms of self-efficacy, positive emotion, and motivation, and comparing gamification-enhanced PR with standard and HAPA-only PR | 24-week three-arm pilot randomized controlled trial with 159 COPD patients (53 per group). Groups: standard PR, HAPA-based PR, and HAPA-gamification-PR. Intervention delivered remotely via online platform; setting was community/home-based. Parallel group design; intervention lasted 12 weeks, followed by 12 weeks observation. Randomization present; blinding status not specified | The application of game design elements and mechanics in a non-game environment | Points, achievement badges, leaderboards, map progress displays, cartoon-style avatars, milestone recognition, visual feedback, rewards, personalized feedback, challenges, progress visualization | The HAPA-gamification-PR group showed significantly higher PR adherence, quality of life, exercise self-efficacy, motivation, and positive affect than both control groups at 12 weeks and maintained improvements at 24 weeks. “Remote gamification PR based on HAPA theory is superior...in improving PR adherence, quality of life, exercise self-efficacy, exercise motivation and positive affect.” Participant feedback highlighted increased engagement and enjoyment. Effect sizes and statistical significance were reported for primary outcomes. Long-term adherence decreased but remained higher than controls | ✓ |
| **Cruz-Cobo et al.** **(34)** |  | To compare the efficacy of an mHealth intervention (eMOTIVA app) versus usual care on compliance with cardiac rehabilitation guidelines, lifestyle, cardiovascular risk factors, and satisfaction in patients with acute coronary syndrome | Randomized controlled clinical trial, parallel group design, 300 patients’ post-percutaneous coronary intervention, hospital and home follow-up, 6 months duration, with blinded data analysis | Not provided | Achievement icons (medals, badges), personalized feedback, reminders, colorful messages, progress graphics, self-comparisons | Favorable results in the intervention group for Mediterranean diet adherence, physical activity, sedentary time, exercise capacity, knowledge, blood pressure, heart rate, and blood sugar. High satisfaction and usability reported | ✓ |

X = negative finding, does not support gamification elements; ✓ = positive finding, supports gamification element.

^a^The majority of outcomes support the use of gamification.
