# Supplementary material for: Practical applications of gamification in patient-centered outcomes research and digital health, and its acceptance in clinical trials
Source: Front Digit Health. 2026 May 29;8:1652217. doi: 10.3389/fdgth.2026.1652217 (PMC13260181; doi:10.3389/fdgth.2026.1652217)
Supplement: Supplementary file 3 [file Table3.docx]

Supplementary Table 3. Heatmap of average rank score per age group

| **Item** | **18–24 years** | **25–34 years** | **35–44 years** | **45–54 years** | **≥55  years** |
| --- | --- | --- | --- | --- | --- |
| Inclusion of mentors | 3.23 | 3.49 | 3.27 | 3.04 | 2.74 |
| Notifications (e.g., reminders, texts) | 3.34 | 3.54 | 3.50 | 3.35 | 3.40 |
| Use of haptic experiences | 3.35 | 3.41 | 3.40 | 2.94 | 2.76 |
| Social competition or comparison (e.g., leaderboards) | 3.35 | 3.40 | 3.29 | 3.14 | 2.59 |
| Inclusion of peer groups (e.g., social) | 3.41 | 3.44 | 3.30 | 3.07 | 2.46 |
| Badges for achievements | 3.51 | 3.61 | 3.57 | 3.26 | 2.82 |
| Use of videos | 3.52 | 3.68 | 3.81 | 3.56 | 3.43 |
| Mini games to play along the way | 3.57 | 3.57 | 3.45 | 3.28 | 2.92 |
| Inclusion of training and education | 3.61 | 3.77 | 3.75 | 3.51 | 3.50 |
| Use of audio | 3.64 | 3.65 | 3.61 | 3.41 | 3.21 |
| Point system or in-game currency with goals | 3.72 | 3.74 | 3.83 | 3.56 | 3.17 |
| Storyline or narrative around a patient journey | 3.74 | 3.80 | 3.77 | 3.50 | 3.19 |
| Quests or challenges | 3.83 | 3.97 | 3.87 | 3.58 | 3.16 |
| Personalization (e.g., avatars) | 3.83 | 3.79 | 3.66 | 3.43 | 2.83 |
| Exploratory or open world approach | 3.86 | 3.92 | 3.83 | 3.63 | 3.31 |
| Customization (ability to choose) | 3.92 | 3.95 | 4.03 | 3.81 | 3.51 |
| Levels and progress feedback | 3.93 | 3.76 | 3.92 | 3.72 | 3.48 |

Survey respondents were asked to select on a response scale from “not at all important” (value = 1) to “extremely important” (value = 5).
