# Supplementary material for: Practical applications of gamification in patient-centered outcomes research and digital health, and its acceptance in clinical trials
Source: Front Digit Health. 2026 May 29;8:1652217. doi: 10.3389/fdgth.2026.1652217 (PMC13260181; doi:10.3389/fdgth.2026.1652217)
Supplement: Supplementary file 4 [file Table4.docx]

Supplementary table 4. Heatmap of average rank per site type

| **Item** | **Private Practice** | **Dedicated Research Clinic** | **Private / Community Hospital** | **Academic Medical Center** |
| --- | --- | --- | --- | --- |
| Storyline or narrative around a patient journey | 2.30 | 2.30 | 2.33 | 2.06 |
| Mini games to play along the way | 2.36 | 2.29 | 2.36 | 2.05 |
| Use of videos (e.g., for training) | 2.43 | 2.23 | 2.18 | 1.88 |
| Use of audio (e.g., for education) | 2.43 | 2.93 | 2.53 | 2.55 |
| Levels and progress feedback | 2.50 | 2.58 | 2.13 | 2.16 |
| Personalization (e.g., use of avatars [in-game representations of patients]) | 2.55 | 2.49 | 2.24 | 2.19 |
| Point system or in-game currency with goals | 2.55 | 2.55 | 2.40 | 2.26 |
| Badges for achievements | 2.70 | 2.58 | 2.33 | 2.16 |
| Use of haptic experiences (e.g., driving using a steering wheel) | 2.73 | 2.83 | 2.58 | 2.45 |
| Inclusion of peer groups (e.g., social networking, collaboration with others) | 2.75 | 2.60 | 2.07 | 2.18 |
| Quests or challenges | 2.77 | 2.30 | 2.13 | 1.80 |
| Social competition or comparison (e.g., leaderboards) | 2.77 | 2.88 | 2.29 | 2.47 |
| Inclusion of training and education within the experience | 2.84 | 2.36 | 2.36 | 2.01 |
| Exploratory or open world approach | 2.84 | 2.73 | 2.47 | 2.26 |
| Inclusion of mentors to support the patients | 3.18 | 2.99 | 2.44 | 2.43 |
| Notifications (e.g., reminders, texts) | 3.18 | 3.06 | 2.67 | 2.48 |
| Customization (ability to choose what elements they want) | 3.18 | 3.18 | 2.78 | 2.66 |

Survey respondents were asked to select on a response scale from “not at all important” (value = 1) to “extremely important” (value = 5).
